# Prohibitin, relocated to the front ends, can control the migration directionality of colorectal cancer cells

**DOI:** 10.18632/oncotarget.19394

**Published:** 2017-07-19

**Authors:** Li-Li Ma, Lan Shen, Gui-Hui Tong, Na Tang, Yang Luo, Li-Li Guo, Chun-Ting Hu, Ying-Xin Huang, Guan Huang, Fang-Yan Jing, Chao Liu, Zhuo-Yi Li, Na Zhou, Qian-Wen Yan, Yan Lei, Shi-Jie Zhu, Zhi-Qiang Cheng, Guang-Wen Cao, Yong-Jian Deng, Yan-Qing Ding

**Affiliations:** ^1^ Department of Pathology, Nanfang Hospital and School of Basic Medical Sciences, Southern Medical University, Guangzhou 510515, China; ^2^ Guangdong Provincial Key Laboratory of Molecular Tumor Pathology, Guangzhou 510515, China; ^3^ Department of Cardiothoracic Surgery, Taishan City People's Hospital, Taishan 529200, China; ^4^ Department of Pathology, Shenzhen People's Hospital, Second Clinical Medical College of Jinan University, Shenzhen 518020, China; ^5^ Department of Urinary Surgery, Nanfang Hospital and the Fifth Affiliated Hospital of Southern Medical University, Guangzhou 510900, China; ^6^ Pathology Center, Shanghai General Hospital Faculty of Basic Medicine, Shanghai Jiao Tong University School of Medicine, Shanghai 200025, China; ^7^ Department of Pathology, Longgang District Central Hospital of Shenzhen, Shenzhen 518116, China; ^8^ Department of Anorectal Surgery, Nanfang Hospital, Southern Medical University, Guangzhou 510515, China; ^9^ Department of Pathology and Laboratory Medicine, Guangdong General Hospital & Guangdong Academy of Medical Sciences, Guangzhou 510080, China; ^10^ Department of Epidemiology, Second Military Medical University, Shanghai 200433, China

**Keywords:** colorectal cancer, migration directionality, prohibitin, vascular endothelial growth factor, cell division cycle 42

## Abstract

Directional migration is a cost-effective movement allowing invasion and metastatic spread of cancer cells. Although migration related to cytoskeletal assembly and microenvironmental chemotaxis has been elucidated, little is known about interaction between extracellular and intracellular molecules for controlling the migrational directionality. A polarized expression of prohibitin (PHB) in the front ends of CRC cells favors metastasis and is correlated with poor prognosis for 545 CRC patients. A high level of vascular endothelial growth factor (VEGF) in the interstitial tissue of CRC patients is associated with metastasis. VEGF bound to its receptor, neuropilin-1, can stimulate the activation of cell division cycle 42, which recruits intra-mitochondrial PHB to the front end of a CRC cell. This intracellular relocation of PHB results in the polymerization and reorganization of filament actin extending to the front end of the cell. As a result, the migration directionality of CRC cells is targeted towards VEGF. Together, these findings identify PHB as a key modulator of directional migration of CRC cells and a target for metastasis.

## INTRODUCTION

Uncontrollable metastases kill 87.5% of patients with distant metastases of colorectal cancer (CRC) within five years of diagnosis [1]. During metastasis, colorectal cancer (CRC) cells break through basement membrane and penetrate into the extracellular stroma with obtaining an enhanced capacity of migration [2]. Since the normal glandular architecture has been destroyed in the invasive adenocarcinoma, the CRC cells can theoretically migrate to the surrounding tissues in all directions. Directional migration may be a cost-effective movement of metastasis [3], but the control of migration directionality is poorly understood. Migration directionality is affected by interactions between elements of the microenvironment including interstitial cells, chemokines, and cancer cells [4]. Vascular endothelial growth factor (VEGF), secreted by the interstitial cells or tumor cells, is one of the important chemokines in the neoplastic microenvironment for angiogenesis, invasion and metastasis [5, 6]. In particular, the VEGF receptor (VEGFR) isoform, neuropilin-1 (NRP1), participates in an autocrine VEGF-dependent signaling mechanism that promotes cancer cell migration [7]. However, mechanisms for VEGF and VEGFR interactions in influencing the migration of cancer cells remain unclear.

Cell polarity is required to establish organization of normal epithelial tissues and restrain cell migration [8]. Apical-basal cell polarity is replaced by front-rear polarity in some metastatic cancers in a process associated with the epithelial-mesenchymal transition [9]. The Rho family GTPases are involved in regulation of the front-rear and apical-basal polarity distributions of plasma membrane and cytoskeletal proteins [10]. Cell division cycle 42 (Cdc42, a member of the Rho family) is activated at the leading-edge of a given cell, which allows the capture and polarization of microtubular orientation by mDia [11] and re-orientation of the Golgi/centrosome to face the direction of migration [12]. Cdc42 activity is involved in invasiveness and metastasis [13]. NRP1 is required for Cdc42 activation, and Cdc42 or NRP1 knockdown impairs sprout migration in primary human endothelial cells [14]. Therefore, the blockage of the intracellular and extracellular proteins involved in front-rear polarity may disrupt the migrating directionality of cancer cells and inhibit metastases.

Prohibitin (PHB, also known as PHB1) is located in the plasma membrane, mitochondria, or nucleus, depending on the cell type and its biological features [15]. PHB is involved in multiple processes such as proliferation, mitochondrial biogenesis, organogenesis, and cell-surface chaperone activities [16]. The deletion of PHB is embryonically lethal to mice and flies [17]. The overexpression of PHB is associated with progression of cancer of the cervix, esophagus, bladder, prostate, and gallbladder [18–22]. Furthermore, PHB locating in plasma membrane promotes metastases [23], and the subcellular location of PHB in cancer cells may be associated with observed biological behaviors, but the underlying mechanism of PHB relocation to the plasma membrane remains unclear.

In this study, we determined that PHB relocated to one end of a CRC cell in a manner that was related to CRC metastasis, and then proposed that PHB relocation may be associated with extra-intracellular interactions. Intriguingly, the polarized distribution of PHB is closely related to the activation of Cdc42 that recruits intra-mitochondrial PHB to the end of a cell facing the direction of extracellular VEGF. Therefore, the polarized distribution of PHB controls the migration directionality of CRC cells in response to extrinsic chemotaxis.

## RESULTS

### Polarized distribution of PHB in CRC is unfavorable for prognosis

**Figure 1:**
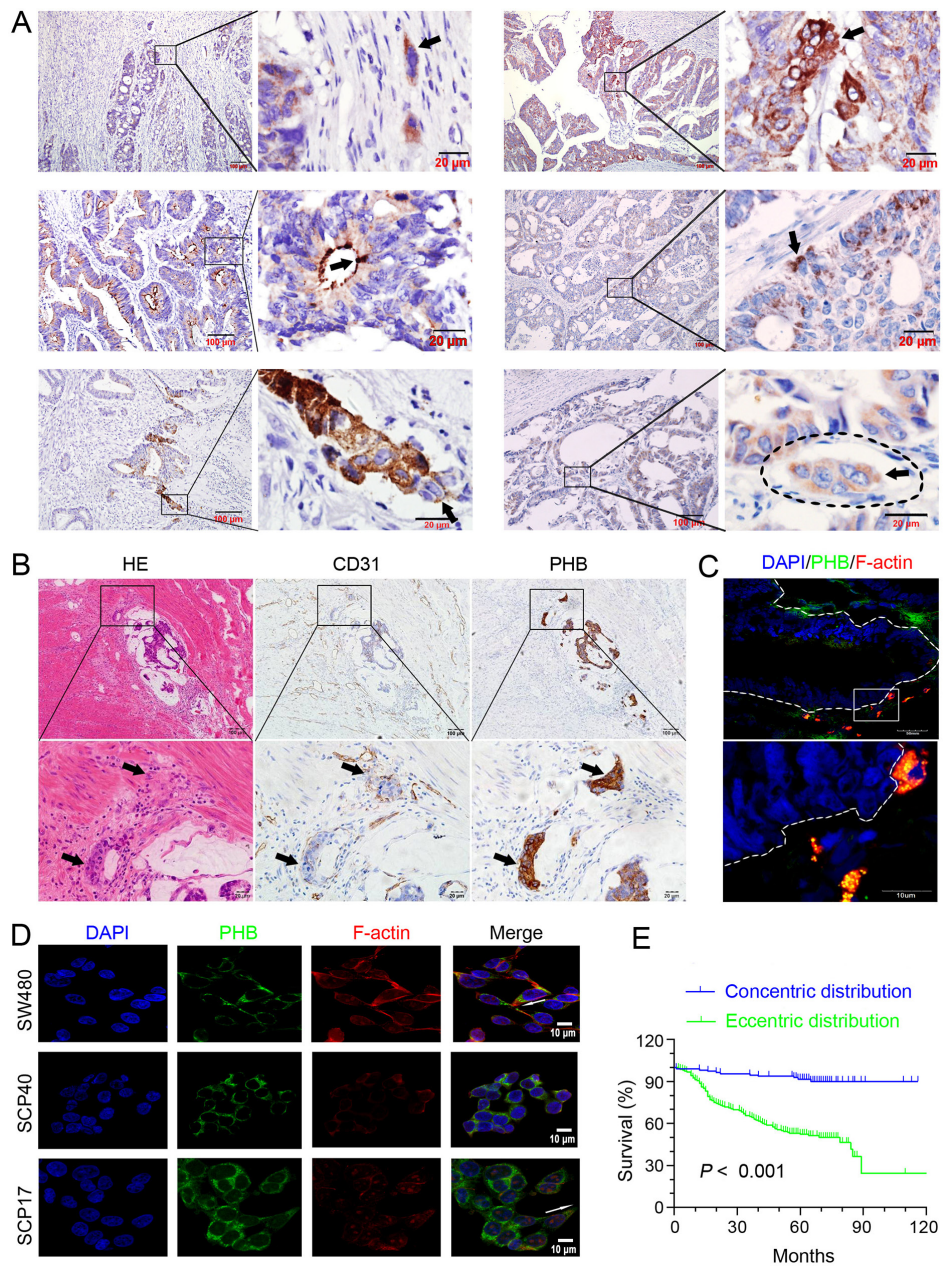
Prohibitin (PHB) expression patterns and prognosis associated with colorectal cancer (CRC) **(A)** PHB was expressed in single and clustered CRC cells (black arrowheads) of moderately and well-differentiated adenocarcinoma (first row); well-differentiated carcinoma with concentric distribution and moderately-differentiated carcinoma with eccentric distribution (second row); clustered cancer cells infiltrating into the stroma and blood vessels that were expressing PHB (third row). Arrowheads indicate the PHB distribution. Scale bars represent 100 μm or 20 μm. **(B)** Images with hematoxylin-eosin staining, CD31, and PHB immunostaining, show intravascular cancer cells in serial tissue sections of CRC. Scale bars represent 100 μm or 20 μm. **(C)** Co-immunostaining for PHB and F-actin in CRC cells migrating out of a cancerous gland profile (white dash line); enlarged image in white frame. Scale bars represent 50 μm or 10 μm. **(D)** Co-immunostaining for PHB and F-actin in CRC cell lines with different metastatic potentials. Arrowheads indicate PHB and F-actin directionality. Scale bars: 10 μm. **(E)** Kaplan-Meier Survival Curve for CRC patients with concentric and eccentric distributions of PHB.

PHB was expressed in single and clustered cells of CRC tissue in 545 patients (Figure 1A, first row). However, PHB expression in CRC tissue was not associated with clinic-pathologic features by qualitative analysis (Table 1). Interestingly, we found that PHB was expressed at one end of a positive cell in well and moderately differentiated adenocarcinomas (Figure 1A; second row), showing polarized expression similar to apical-basal polarity. Poorly differentiated adenocarcinoma samples were excluded since there were no intact glands for reference (Supplementary Figure 1). We classified all samples that were positive for PHB into two groups: those with concentric expression of PHB (apical), and those with eccentric expression (basal) (Figure 1A; second row). The cancer cells with PHB expression embedded in or invading the extracellular matrix exhibited front-rear polarity. In addition, single or clustered CRC cells invading the stroma (Figure 1A; first and third row) or the vasculature (Figure 1A; third row, Figure 1B) were observed together with front-rear polarity of PHB expression. The subcellular location of PHB showed polarized distribution in CRC cells and seems to control the directionality of the migrating cell.

**Table 1:** Relationship between PHB immunoreactivity and clinicopathologic features in patients with CRC (n = 545)

Characteristic	No. patients %	PHB immunoreactivity		*P* value
		Positive *n* = 436 (%)	Negative *n* = 109 (%)	
Gender				0.190
Male	325 (59.6)	254 (58.3)	71 (65.1)	
Female	220 (40.4)	182 (41.7)	38 (34.9)	
Age(yrs)				0.797
≤ 60	279 (51.2)	222 (50.9)	57 (52.3)	
> 60	266 (48.8)	214 (49.1)	52 (47.7)	
Location				0.731
Colon	258 (47.3)	208 (47.7)	50 (45.9)	
Rectum	287 (52.7)	228 (52.3)	59 (54.1)	
Histo. differ.				0.068
Well	190 (34.9)	156 (35.8)	34 (31.2)	
Moderate	247 (45.3)	203 (46.6)	44 (40.4)	
Poor	55 (10.1)	41 (9.4)	14 (12.8)	
Mucinous	53 (9.7)	36 (8.2)	17 (15.6)	
TNM tumor stage				0.074
I	64 (11.7)	47 (10.8)	17 (15.6)	
II	227 (41.7)	180 (41.3)	47 (43.1)	
III	243 (44.6)	198 (45.4)	45 (41.3)	
IV	11 (2.0)	11 (2.5)	0 (0.0)	
LN metastasis				0.280
Negative	356 (65.3)	280 (64.2)	76 (69.7)	
Positive	189 (34.7)	156 (35.8)	33 (30.3)	
Dis. metastasis				0.862
Negative	314 (57.6)	252 (57.8)	62 (56.9)	
Positive	231 (42.4)	184 (42.2)	47 (43.1)	
Status				0.657
Censored	345 (63.3)	278 (63.8)	67 (61.5)	
Death	200 (36.7)	158 (36.2)	42 (38.5)	
Survival time				0.898
≤ 60 months	263 (48.3)	211 (48.4)	52 (47.7)	
> 60 months	282 (51.7)	225 (51.6)	57 (52.3)	

LN = lymph node; Dis. metastasis = distant metastasis; Histo. differ. = histopathological type and differentiation.

Significant differences were observed between concentric and eccentric groups in differentiation (*P* < 0.01), survival time (*P* < 0.001), TNM stage (*P* < 0.001), and lymph node (*P* < 0.05) or distant metastases (*P* < 0.001), but not in age, sex, or tumor sites (Table 2). Interestingly, co-localization was observed by immunostaining for PHB and filamentous actin (F-actin) in CRC cells that had migrated beyond the gland profile (Figure 1C). This pattern was also observed in SCP17 (a high metastatic sub-line of SW480 CRC cells), SCP40 (a low metastatic sub-line of SW480 cells, as described in our previous research [24]), and SW480 cells (Figure 1D). The co-staining of PHB and F-actin showed more co-localization in the cell ends of SCP17 than in SCP40 (Figure 1D). Kaplan-Meier survival curves based on 11 years of follow-up data after radical surgery showed unfavorable prognosis for patients with eccentric expression (*P* < 0.001, Figure 1E). Thus, cancer cells with eccentric expression of PHB were associated with an unfavorable prognosis, indicating that PHB with eccentric expression promoted aggressive behaviors of CRC cells.

**Table 2:** PHB with concentric and eccentric distributions of CRC patients in association with clinicopathologic charcteristics (*n* = 272)

Characteristic	No. patients %	Direction of PHB		*P* value
		CON, *n* = 112 (%)	ECC, *n* = 160 (%)	
Gender				0.812
Male	165 (60.7)	67 (59.8)	98 (61.3)	
Female	107 (39.3)	45 (40.2)	62 (38.7)	
Age(yrs)				0.965
≤ 60	121 (44.5)	50 (44.6)	71 (44.4)	
> 60	151 (55.5)	62 (55.4)	89 (55.6)	
Location				0.816
Colon	141 (51.8)	59 (52.7)	82 (51.3)	
Rectum	131 (48.2)	53 (47.3)	78 (48.7)	
Differentiation				0.009
Well	120 (44.1)	60 (53.6)	60 (37.5)	
Moderate	152 (55.9)	52 (46.4)	100 (62.5)	
TNM tumor stage				0.000
I	32 (11.8)	22 (19.6)	10 (6.3)	
II	125 (45.9)	56 (50.0)	69 (43.1)	
III	109 (40.1)	33 (29.5)	76 (47.5)	
IV	6 (2.2)	1 (0.9)	5 (3.1)	
LN metastasis				0.024
Negative	191 (70.2)	87 (77.7)	104 (65.0)	
Positive	81 (29.8)	25 (22.3)	56 (35.0)	
Dis. Metastasis				0.000
Negative	167 (61.4)	101 (90.2)	66 (41.2)	
Positive	105 (38.6)	11 (9.8)	94 (58.8)	
Status				0.000
Censored	180 (66.2)	102 (91.1)	78 (48.7)	
Death	92 (33.8)	10 (8.9)	82(51.3)	
Survival time				0.000
≤ 60 months	131 (48.2)	34 (30.4)	97 (60.6)	
> 60 months	141 (51.8)	78 (69.6)	63 (39.4)	

LN=lymph node; Dis. metastasis = distant metastasis; CON = concentric distribution;ECC = eccentric distribution.

### In response to VEGF, PHB in the front end of a CRC cell determines the directionality of migration

**Figure 2:**
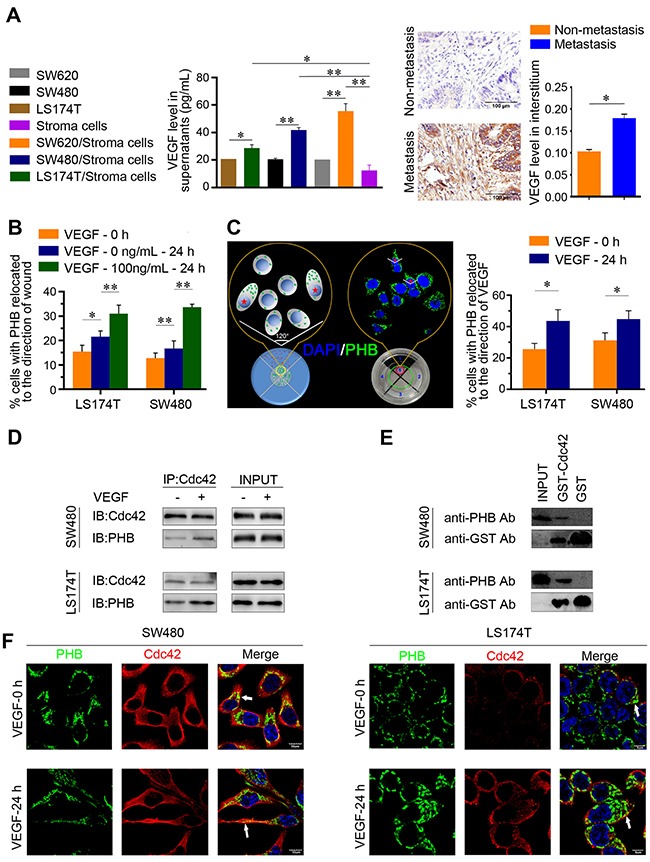
The intracellular relocation of PHB was induced by vascular endothelial growth factor (VEGF) in CRC cells **(A)** Stromal cells were mixed with LS174T or SW480 or SW620 cells and were cultured for 24 h. Supernatants were collected and VEGF levels were determined using the enzyme-linked immune sorbent assay (ELISA). *P < 0.01, **P < 0.001. Data are shown as means ± SD. Levels of VEGF expression in the interstitial tissue are shown in primary CRC with metastasis and non-metastasis. *P < 0.001. Data are shown as means ± SEM. **(B)** Quantitative analysis of wound-healing assays was performed by calculating the percentage of cells in which PHB was relocated to the direction of wound. *P < 0.01 and **P < 0.001. Data are shown as means ± SD. **(C)** A schematic model and an experimental example for the polarized migration assay. A mixture of VEGF and Matrigel was placed in area 1, Matrigel alone was placed in area 2, 3, and 4, and the cells in area 5 were chosen for polarization analysis. Cells in which PHB was located within the 120° angle were counted as being in the direction of VEGF stimulation, and are marked as red stars. The quantitative analysis of polarity assays was performed by calculation of the percentage of cells in which PHB was relocated to the direction of VEGF stimulation. *P < 0.001 compared with VEGF treatment for 0 h. Data are shown as means ± SD. **(D)** Co-immunoprecipitation assay with Cdc42. Cdc42 and PHB were expressed in SW480/LS174T with (+) or without (-) VEGF (100 ng/mL) treatment for 24 h. **(E)** Indicated GST-fusion proteins were incubated with lysates from SW480/LS174T and precipitated with glutathione beads. PHB was detected in the eluates of GST-Cdc42. **(F)** Co-immunostaining for PHB and Cdc42 in SW480/LS174T with or without VEGF stimulation. The arrowheads indicate PHB and Cdc42 directionality. Scale bars: 10 μm.

VEGF (also VEGF165, a variant of VEGF-A) is secreted by various cells including cancer cells, endothelial cells, and vascular smooth muscle cells. In the tumor microenvironment, stromal cells play a key role in metastasis. Secreted VEGF was measured in several types of stromal cells, including human umbilical vein endothelial cells (HUVECs), peripheral blood mononuclear cells (PBMCs), and fibroblasts. After culturing for 24 h, VEGF was not detected in the media of individual stromal cells. When the CRC cell lines of SW620, SW480, and LS174T were co-cultured with the above-mentioned stromal cells, respectively, VEGF was strongly detected in the media. VEGF level was the highest in the SW620 mixture among them (Figure 2A). VEGF is overexpressed in several cancer types and is related to growth and invasiveness [25]. Interstitial VEGF in primary CRC tissues with metastases were expressed to higher levels than in those with non-metastases (Figure 2A). These indicated that the mixed cells of stroma and cancer mimicked the wild microenvironment of cancer tissues. The VEGF in the supernatant was analogous to the VEGF of cancer interstitia which was detected in human CRC tissues using immunohistochemistry examination. Thus, interactions between cancer cells and stromal cells appeared to favor VEGF secretion.

Cancer metastases share chemoattractant-directed migration through blood vessels to distant organs and tissues [4]. Given that VEGF may play a role in relocating PHB, a wound-healing assay was performed, and the cells expressing PHB within the angle of 120° facing the wound were counted (Supplementary Figure 2A), the angle of 120° is accordance with the method of Etienne-Manneville S and Hall A described [26]. After VEGF stimulation for 24 h, the percentage of SW480 and LS174T cells with PHB expression relocated to the wound was significantly increased (Figure 2B). We then established a polarity model with Matrigel to identify the directionality of migrating cells (Figure 2C). VEGF was fixed in semi-solid Matrigel in the direction of stimulation to determine the directionality of migrating cells. Only the cells in which PHB relocated within an angle of 120° were considered as showing a reaction to VEGF stimulation. The direction of PHB relocation showed time-concentration stimulation (Supplementary Figure 2B and 2C). However, the Matrigel concentration had no effect on PHB relocation (Supplementary Figure 2D). After stimulation by VEGF for 24 h, more CRC cells showed PHB relocation than the controls (Figure 2C, Supplementary Figure 2E). Thus, extrinsic VEGF stimulation promotes the relocation of PHB to one end of a CRC cell.

In polarized migration cells, Cdc42 localizes to the leading edge of the cells [26]. Co-immunoprecipitation (Co-IP) analysis showed more endogenous PHB precipitated with the Cdc42 in the VEGF stimulation group (Figure 2D). To examine whether this interaction is direct, we next performed a binding assay *in vitro* using purified GST-Cdc42 and found that PHB interacted with GST-Cdc42 (Figure 2E). Double immunostaining showed that the polarized expression of PHB and Cdc2 was more obvious in the VEGF stimulation group (Figure 2F). Overall, these data indicate that Cdc42 and PHB form a complex at the front end of a CRC cell, and the subcellular location of PHB seems to control the directionality of migrating cells.

### VEGF/NRP1 binding can activate Cdc42 to recruit PHB

**Figure 3:**
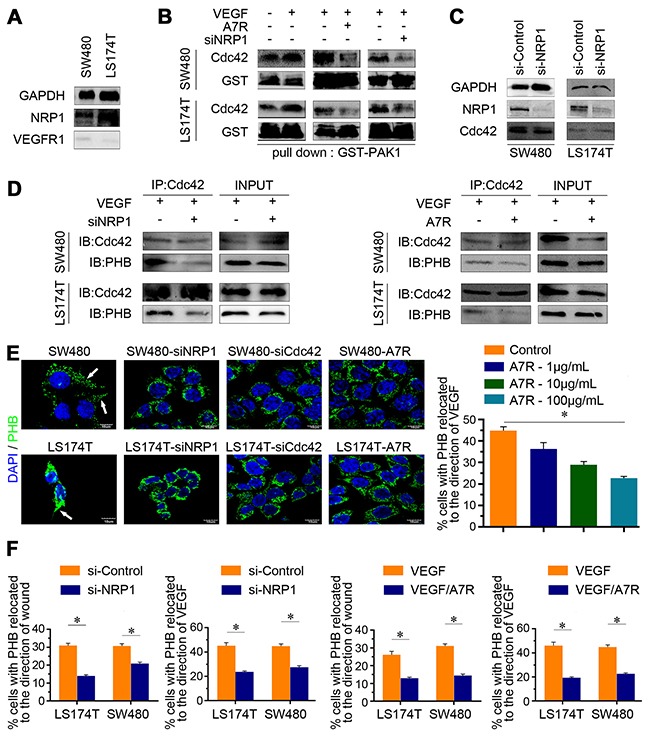
Polarized distribution of PHB and the recruitment of activated Cdc42 **(A)** Western blot analysis showed that CRC cells express VEGF receptors (VEGFR) as NRP1 and as VEGFR1 in LS174T and SW480 cells. **(B-C)** SW480/LS174T were starved overnight in 2% FBS and then either untreated (-) or treated (+) with 100 ng/mL VEGF for 24 h; SW480/LS174T were cultured with 100 ng/mL VEGF and either untreated (-) or treated (+) with A7R for 24 h; SW480/LS174T were transfected with control (-) or NRP1 siRNA (+) and then cultured with 100 ng/mL VEGF. The lysates were obtained and Cdc42-GTP levels were assessed by PAK1 pull-down assay. **(D)** Co-immunoprecipitation assay with Cdc42. Cdc42 and PHB were expressed in SW480/LS174T with control (-) or NRP1 siRNA (+) and without (-) or with supplemented A7R (+) treatment for 24 h. **(E)** PHB staining of SW480/LS174T, SW480/LS174T with siRNA of NRP1 (SW480-siNRP1, LS174T-siNRP1) or Cdc42 (SW480-siCDC42, LS174T-siCDC42) or A7R (SW480-A7R, LS174T-A7R). Scale bar, 10 μm. The percentage of cells with relocated PHB was negatively associated with A7R concentration. *P < 0.001. Data are shown as means ± SEM. **(F)** Wound-healing and polarity assays of siControl/siNRP1 and VEGF-inhibited (A7R) CRC cells, in which PHB was relocated to the direction of a wound within 200 μm or the direction of VEGF stimulation. Data are shown as means ± SEM. *P < 0.001.

Preclinical studies have shown that NRP1 is required induction by the extracellular matrix of Cdc42 activation in primary human endothelial cells [14]. In our study, we detected the expression levels of VEGF receptors. CRC cell lines expressed NRP1 and minimal VEGFR1 (Figure 3A), but did not express NRP2 or VEGFR2 (data not shown). A pull-down assay with the p21-activated protein kinase PAK1 was used to measure GTP-bound Cdc42 and showed that Cdc42 can be activated by VEGF (Figure 3B and 3C). However, when NRP1 was silenced (Figure 3C) or blocked with ATWLPPR (A7R, a reagent has been shown to inhibit VEGF/NRP-1 [27].), Cdc42 activation was reduced (Figure 3B) and endogenous PHB precipitated with Cdc42 was also decreased, even with VEGF stimulation (Figure 3D). These results suggest that the engagement of VEGF and NRP1 is sufficient for Cdc42 activation.

Additionally, fewer cells with polarized distribution of PHB were observed when NRP1 or Cdc42 (Supplementary Figure 3) was decreased or when the experiment was performed using media supplemented with 100 μg/mL A7R (Figure 3E). The results of the wound-healing assay and the polarity model also showed that the PHB relocation capacity correlated with the levels of Cdc42 activation (Figure 3F). Taken together, our data revealed that VEGF/NRP1 binding first activated Cdc42, which triggered the relocation of PHB.

### PHB relocation depends on microtubule transportation

**Figure 4:**
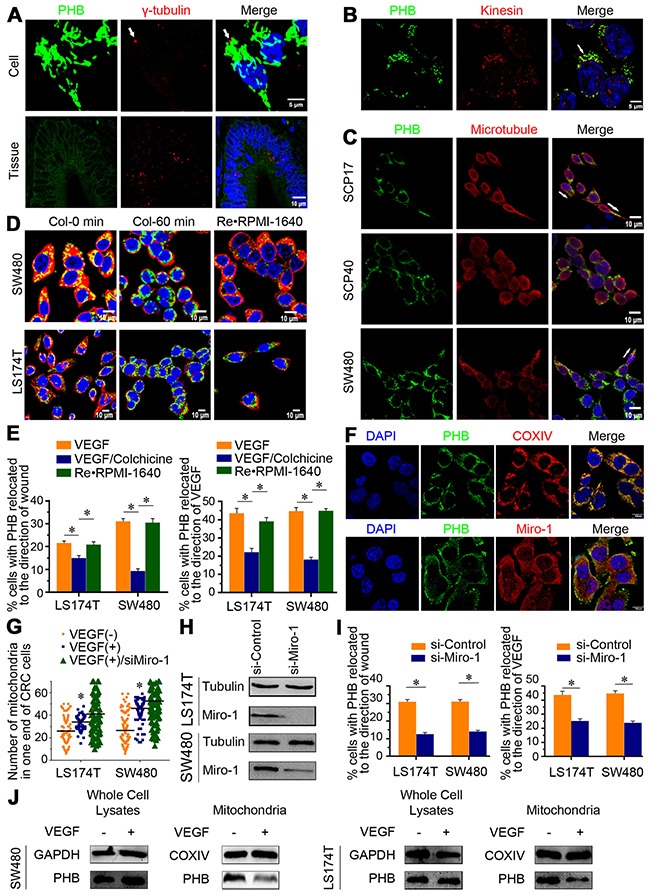
The relocation of PHB to one cellular end is associated with microtubule transportation and may have originated from mitochondrial release **(A)** Immunostaining for PHB and γ-tubulin in CRC cell lines and tissues, where the white arrow indicates γ-tubulin. Scale bar: 5 μm and 10 μm. **(B)** PHB and kinesin (a motor for transportation) were co-localized in CRC cells. Scale bars: 5 μm. **(C)** PHB and microtubules were co-immunostained in CRC cell sublines that differed in metastatic potential. White arrows indicate the directionality of PHB and microtubules. Scale bar: 10 μm. **(D)** PHB and microtubule staining of cells without (Col-0 min) or with colchicine treatment (Col-60 min), and then colchicine was replaced by RPMI-1640 (RE·RPMI-1640). Scale bar, 10 μm, col = colchicine. **(E)** Wound-healing assays of CRC cells in which PHB relocated to the direction of the wound. Polarity assays of CRC cells in which PHB was relocated to the direction of VEGF stimulation. Data are shown as means ± SEM. *P < 0.001. **(F)** PHB/COXIV and PHB/Miro-1 co-expressed in CRC cells. Scale bar: 10 μm. **(G)** Scatter plot graph showed that mitochondria were concentrated on one end of the CRC cells following VEGF stimulation. When Miro-1 was depleted, the concentrated distribution of mitochondria was impaired (VEGF(+)/siMiro-1). Data are shown as means ± SD, *P < 0.001. **(H)** Western blot analysis of control CRC cells (si-Control) and Miro-1-silenced cells (siMiro-1). **(I)** Quantitative analysis of wound-healing assays performed by calculating the percentage of cells within 200 μm of the wound; PHB was relocated in the direction of the wound after VEGF stimulation. Quantitative analysis of polarity assays was performed by calculating the percentage of cells, in which PHB relocation faced the direction of VEGF stimulation. Data are shown as means ± SEM. *P < 0.001. **(J)** Whole-cell lysates and mitochondrial fractions were isolated and probed for PHB.

The microtubule organizing center (MTOC) or centrosome reorients to a location in front of the nucleus, toward the direction of cell migration [28]. The MTOC position was more irregular in cancer cells than normal cells, a property that is associated with poor prognosis [29]. γ-tubulin (one of the MTOC components) and PHB are located in the same direction of CRC cells and tissue (Figure 4A). Microtubules serve as highways for the intracellular transportation of various cellular components throughout the cell by motor proteins, including organelles, vesicles, proteins, and signaling molecules [30]. Anterograde cargo movement is driven by kinesin, and we also observed that kinesin and PHB are co-localized in CRC cells (Figure 4B). Studies have shown that the microtubules provide cortical polarity in cells [31] and that the localized activation of Cdc42 controls the polarity of microtubule cytoskeletons [11]. PHB attached to microtubules was observed in SCP17, SCP40, and SW480 CRC cells (Figure 4C). Moreover, concentrated foci of PHB were more easily observed in the high metastatic potential sub-line of SW480 (SCP17) than in the low metastatic potential sub-line (SCP40) in accordance with the polarity of the microtubular architecture.

In addition to VEGF stimulation, the cells were exposed to 10 ng/mL colchicine to inhibit tubulin assembly and microtubule formation. When cells were exposed to colchicine for 60 min, PHB disappeared from the front ends of the CRC cells (Figure 4D). After 60 min, there were fewer cells expressing PHB in the front ends of cells that were oriented towards the direction of the wound or the VEGF stimulation (Figure 4E). However, PHB reappeared in the front ends after the removal of colchicine, with PHB again localized towards the wound or the direction of VEGF stimulation (Figure 4D and 4E). These data revealed that polarized aggregation of PHB in the front was associated with microtubule transportation.

### PHB that is relocated to the front ends, originates from mitochondria

The interaction of Miro-1 with kinesin is reported to promote anterograde mitochondrial movement along the microtubules. Miro-1 is anchored in the mitochondrial membrane and is necessary for mitochondrial movement. [32]. Our data showed that PHB is mainly located in the mitochondria (COXIV, a mitochondrial marker, Figure 4F). PHB localized to the leading-edge of cells due to microtubule transportation, and the co-localization of Miro-1 and PHB showed that Miro-1 forms a bridge between the intra-mitochondrial PHB and kinesin (Figure 4F). Consistent with the polarized distribution of PHB, mitochondria were also concentrated at one end of a CRC cell after VEGF stimulation (Figure 4G). Intra-mitochondrial PHB relocated to the front dependent on the interaction between Miro-1 and kinesin.

To explore the origin of PHB localization in the front ends of the CRC cells, specific siRNA deletion of Miro-1 was used (Figure 4H). As expected, Miro-1-depleted cells presented an impaired ability to concentrate mitochondria and relocate PHB to any ends of cells, even in the presence of VEGF (Figure 4G-4I). Thus, the lack of Miro-1 prevents front-end relocalization of intra-mitochondrial PHB. PHB immunostaining was next performed in isolated mitochondria proteins, with and without VEGF stimulation. In the presence of VEGF, PHB was significantly decreased in the mitochondrial fraction, but no changes of protein levels in PHB were observed in the whole-cell lysates (Figure 4J). These data suggest that the reorientation of PHB occurs in response to mitochondrial release after VEGF stimulation.

### Subcellularly polarized relocation of PHB depending on VEGF stimulation

**Figure 5:**
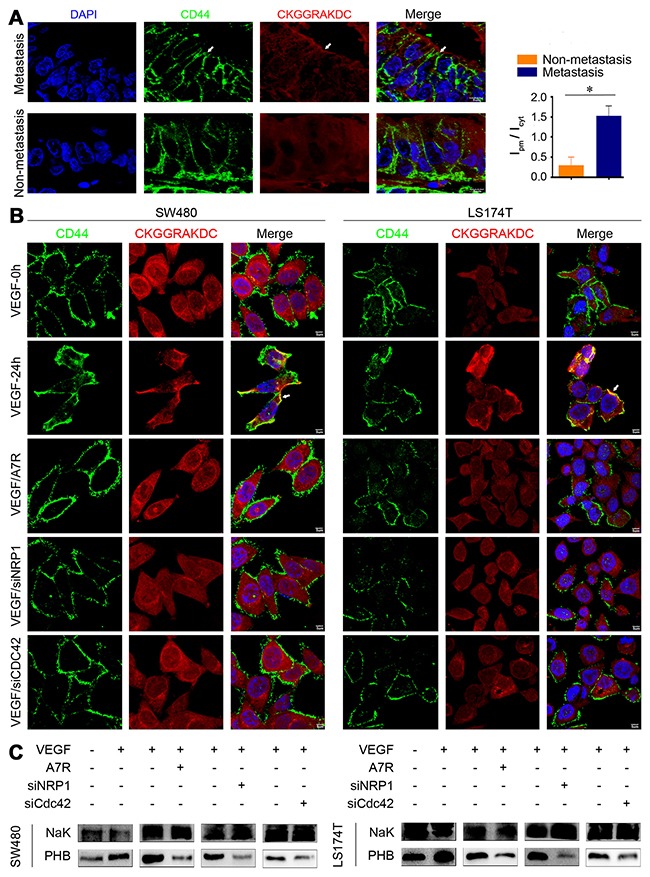
PHB relocation together with the recruitment of activated Cdc42 in the cytoplasma membrane **(A)** Immunostaining for CKGGRAKDC-Rhodamine and CDC44 in tissues. CKGGRAKDC-Rhodamine was specifically linked to the PHB peptide motif. Positive staining of CKGGRAKDC-Rhodamine in the cytoplasma of primary CRC without metastasis (non-metastasis) or in the cytomembranes of primary CRC with metastasis (metastasis). Scale bar: 5 μm. **(B)** CKGGRAKDC-Rhodamine/CD44 stained SW480/LS174T, SW480/LS174T were treated as follows: SW480/LS174T were starved overnight in 2% FBS (VEGF-0h or Control); SW480/LS174T were starved overnight in 2% FBS and treated with 100 ng/mL VEGF for 24 h (VEGF-24h or VEGF); SW480/LS174T were cultured with 100 ng/mL VEGF mixed with A7R for 24 h (VEGF/A7R or A7R); SW480/LS174T were transfected with NRP1 siRNA and then cultured with 100 ng/mL VEGF (VEGF/siNRP1 or siNRP1), and SW480/LS174T were transfected with Cdc42 siRNA and then cultured with 100 ng/mL VEGF (VEGF/siCDC42 or siCDC42). Scale bar: 5 μm. **(C)** Cell membrane fractions were isolated and probed for PHB.

The relocation of PHB from the cytoplasm to the cell surface is critical for drug resistance, and PHB in the plasma membrane is associated with the metastasis of cancer cells [23]. To explore the relationship between PHB-location and metastasis, we first analyzed the distribution of plasma membrane PHB in primary cancer with metastasis or non-metastasis of CRC tissues. For this analysis, we used a CKGGRAKDC-rhodamine peptide (which can specifically bind PHB) [33] to co-immunostaining with CD44 (an intramembrane protein marker). We found that the PHB level was significantly higher in the plasma membranes of primary CRC with metastasis than non-metastasis (Figure 5A).

PHB is located on the plasma membrane of CRC cells and was visualized using the CKGGRAKDC-rhodamine peptide after VEGF stimulation. The PHB located to the plasma membrane was impaired in cells supplemented with A7R or in NRP1/Cdc42-silenced cells (Figure 5B). PHB immunostaining was performed in the fraction of isolated proteins of cell surface. PHB was significantly increased in the membrane fraction of VEGF-stimulated cells, but was markedly reduced after knocking down Cdc42/NRP1 or blocking with A7R (Figure 5C). These data indicated that activated Cdc42 recruits PHB to the cytoplasma membrane after VEGF stimulation.

### Polarized relocation of PHB controlled the directionality of F-actin extension and CRC cell migration

**Figure 6:**
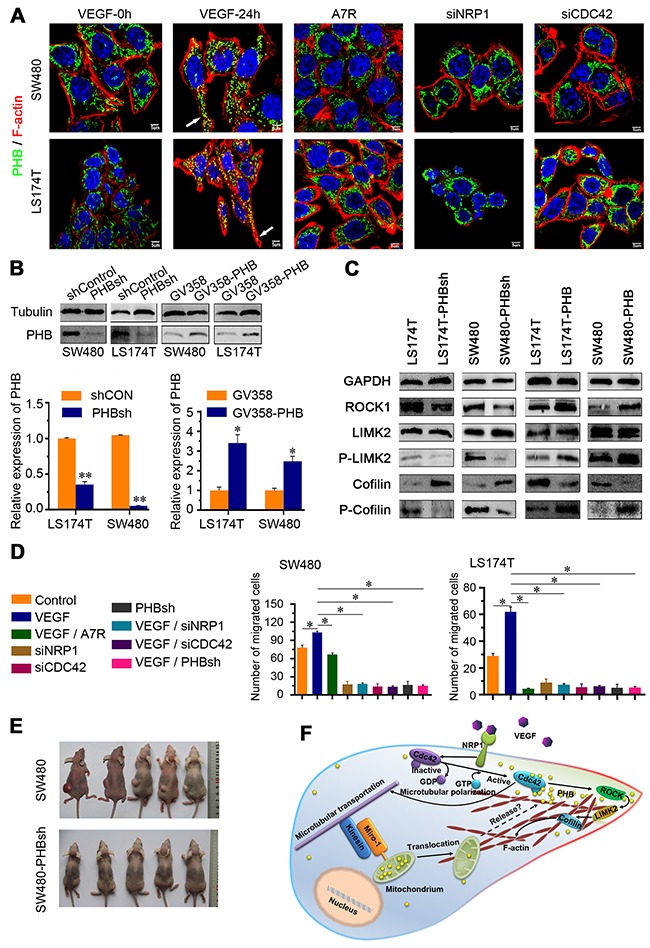
PHB with polarized distribution regulates cytoskeletal remodeling and directional migration of CRC cells **(A)** Immunofluorescence image of F-actin and PHB in CRC cells (VEGF-0h, VEGF-24h, A7R, siNRP1, and siCDC42). The white arrows indicate the polarity of PHB and F-actin and were the most prominent in the VEGF stimulation group. Scale bar: 5 μm. **(B)** Western blot analysis of PHB in control (shControl)/PHB knockdown (PHBsh) cells, and in GV358(control)/GV358PHB(PHB-up-regulated)-transfected cell. Real-time quantitative polymerase chain reaction for amplification of PHB was measured in the above-mentioned cells Error bars represent the mean ± SD of triplicate experiments; *P < 0.01. **(C)** Different CRC cell lines were immunostained for PHB and the expression of cofilin, phosphorylated-cofilin (p-cofilin), ROCK1, LIMK2, and phosphorylated-LIMK2 (p-LIMK2). **(D)** Quantitative analysis results of the transwell assay of CRC cells. Data are presented as the means ± SD from triplicate experiments. *P < 0.001. **(E)** Subcutaneous implantation of SW480/SW480-PHBsh cells in the nude mice. **(F)** Schematic of PHB controlling the directionality of migration of cancer cells. VEGF/NRP1 engagement actives Cdc42, which recruits intra-mitochondrial PHB to the leading edge. Miro-1/Kinesin binding participates in the translocation of mitochondrium which attaches to microtubles. PHB is released to the leading edge to control F-actin extension and the directionality of migration.

Cytoskeletal signaling regulates several cell processes, including polarity and movement. Microfilaments consist of fibrous polymers of F-actin and are major components of the cytoskeleton. We found that the co-stained PHB and F-actin with polarized distribution were more prominent in the cells that were cultured with VEGF-conditioned media, but were not observed in A7R supplemented media or NRP1/Cdc42 silenced cells (Figure 6A). In addition, PHB and F-actin were polarizedly co-localized to the cell projections, which are more abundant in SCP17 cells than in the PHB-silenced cells. Correspondingly, when PHB was overexpressed in SCP40 cells, F-actin polarized expression was increased (Supplementary Figure 4A).

Dynamic remodeling of the F-actin cytoskeleton is important for migration of cancer cells [34]. We wondered whether PHB expressed to higher levels in the front ends of cells could induce CRC cell migration through the phosphorylation of cofilin (p-cofilin) to strengthen F-actin polymerization via LIM-kinase (LIMK). This model was tested in LS174T and SW480, cells that moderately expressed PHB (Supplementary Figure 4B). When PHB was downregulated (Figure 6B), p-cofilin expression was weakened (Figure 6C). When PHB was overexpressed (Figure 6B), the p-cofilin signal was stronger (Figure 6C). These indicated that polarized PHB preferentially participated in cofilin/F-actin cytoskeletal remodeling and F-actin extension (Figure 6A), and controlled the directionality of migration of CRC cells (Figure 1C and 1D). The strengthened migration capacity of cancer cells benefited from VEGF stimulation was not observed in PHB/NRP1/Cdc42 silenced cells or A7R-conditioned media (Figure 6D, Supplementary Figure 4C). When SW480 cells or shPHB/SW480 cells were subcutaneously implanted into nude mice, SW480 cells produced tumor masses but shPHB/SW480 did not (Figure 6E), which was also shown in the animal model of the cecal-orthotopic tansplantation (data not shown). However, the growth curve showed that the growth ability of PHBsh tumor cells is not inhibited in culture (*P* < 0.01), conversely, PHBsh tumor cells showed a bit of high growth after cultured for 4 days (Supplementary Figure 4D). It is in accordance with Guo et al [35]. Probably, the bioactivities of PHB in cancer cells might be associated with microenvironment factors. These results indicate that polarized distribution of PHB dominates the directionality of F-actin extension, supporting the directional migration of the CRC cells.

## DISCUSSION

Interstitial invasiveness and the circulatory infiltration of primary cancer favor distant metastasis, but directional migration enhances the efficiency of cancer cell delivery [3]. The tumor microenvironment, including cytokines, chemokines, growth factors, and matrix remodeling proteins, can significantly affect the directional migration of cancer cells [4]. However, there is little published data to explain the mechanism of the migrating directionality of a cancer cell. Previous studies have shown that VEGF mainly functions as an angiogenic factor, stimulating neoangiogenesis and increasing vascular permeability, as occurs in cancer treatment [36]. However, our study showed an additional role for VEGF, and our results show that VEGF expression stimulated the relocation of PHB to the front ends of CRC cells to subsequently control the directionality of migration (Figure 6F).

PHB is over-expressed in various types of cancers [18]. Although high expression of PHB in breast cancer inhibits cell growth [37], PHB expression levels showed no relationship with prognosis or metastasis of CRC in our study. Surprisingly, we found unique patterns of PHB expression with eccentric and concentric distributions in cancerous glands using immunohistochemical examination, and further analysis demonstrated that the eccentric distribution was closely linked to metastasis and poor prognosis for CRC patients. Furthermore, the eccentric distribution of PHB located at the front end of a CRC cell may infiltrate the interstitial tissue. The orientation indicates the directionality of cancer cell migration. Therefore, we should explore the polarized distribution of PHB that results from pathogenic changes.

Cell polarity is produced by subcellular asymmetric compartmentalization or the directional transportation of proteins or organelles, ultimately leading to cell type-specific morphological architecture [31]. Cdc42 controls polarity via its effector aPKC, which leads to MTOC reorientation and microtubule capture at the leading edge in fibroblasts [11, 12]. Activated Cdc42 promotes the relocation of distinct molecular complexes to the leading edge of cells, allowing the capture of microtubule plus ends and leading to the polarization of the microtubule cytoskeleton [38]. Persistent directional transportation may be influenced by interactions between internal and external environmental factors of CRC cells, such as integrin, fiber adhesion proteins, polysaccharides, epidermal cell growth factors, VEGF, and other [39]. Here, we found that extracellular VEGF bound to NRP1 in the cancer cell membrane, which activated Cdc42 to recruit intra-mitochondrial PHB to the front ends of CRC cells. In addition, Miro-1 and kinesin connections mediated mitochondrial translocation through microtubular transportation, releasing intra-mitochondrial PHB to the front ends. However, the details of this intra-mitochondrial PHB release remain to be elucidated.

Previous reports have suggested that PHB is involved in metastasis via activation of the Ras-C-Raf-MEK-ERK pathway, modulation of TGF-β signaling, or transcriptional regulation [40, 41]. Our data show that PHB positioned in the front end of the plasma membrane not only acts as a positive regulator of the ROCK/LIMK pathway by altering the expression of p-cofilin in CRC cells, but also weakens the depolymerization of F-actin and extends the F-actin to the leading-edge. *in vivo* model should be more intuitive to show the role of PHB to control the migration directionality of cancer cells. Since SW480-PHBsh cells were unable to produce tumors in nude mice, just like PHB-deletion of embryonically lethal to mice in the findings of McClung, JK. et al [17], the vivo model was unachievable. In summary, PHB can control the directionality of migration in CRC cells.

In conclusion, this study described one aspect of cancer metastasis. The relocation of intracellular PHB to the front ends of CRC cells can affect the directionality of migration by microenvironmental stimulation, which may enhance the effectiveness of metastasis. Overall, these findings and future analyses are theoretically and practically relevant to target therapeutics for tumor metastasis.

## MATERIALS AND METHODS

### Cell culture

Human CRC cell lines (SW480, LS174T, and SW620) and peripheral blood mononuclear cells (PBMCs) were cultured as described in Wu et al [42]. Fibroblasts were cultured as described in Xu et al [43]. HUVECs were maintained in DMEM/F-12 (GIBCO, Shanghai, china). Cells were maintained by the Guangdong Provincial Key Laboratory of Molecular Tumor Pathology, Guangzhou, China. All relevant human cell lines used in experiments were obtained from ATCC, which authenticates using short tandem repeat profiling.

### VEGF detection by ELISA

Human VEGF (VEGF165) Quantitative ELISA Kits (Cloud-Clone Corp.) were used according to the manufacturer's instructions. Approximately 100 μL of conditioned media, in which HUVECs, PBMCs, and fibroblasts, and SW620, SW480, and LS174T CRC cells were cultured in random combinations, were collected from triplicate samples.

### Western blot

LS174T and SW480 cells were seeded in 6-well plates. Cell extracts were prepared in ice-cold lysis buffer containing protease inhibitor. The separated proteins were transferred to polyvinylidene fluoride (PVDF) membranes (Millipore) and further incubated with specific antibodies including PHB (1:500, EP2803Y), F-actin (5 μg/mL), Neuropilin1 (1:400, EPR3113, Abcam), RhoT1 (1:50), p-cofilin (1:100, P23528, ABclonal) and antibodies purchased from Cell Signaling Technology such as cofilin (1:500, D3F9), ROCK1 (1:500, C8F7), LIMK2 (1:500, 8C11), and p-LIMK2 (1:500, Thr505). Following incubated with the primary antibodies and the matched secondary antibodies, protein bands were visualized with ECL reagent (Thermo Scientific Inc.).

### Immunoprecipitation

The CRC cells were stimulated with or without 100 ng/mL VEGF for 24 h and then lysed. The cell lysates were incubated with antibodies to Cdc42 (1-2 μg/100-500 μg of total protein, sc-8401, Santa Cruz) at 4 °C overnight. Protein A/G PLUS-Agarose (Santa Cruz) was added and rotated at 4 °C for 1.5 h. After washing the beads, proteins were analyzed by SDS-PAGE and western blotting. In the input analysis, 1/10 volume of the cell lysate was used.

### Immunohistochemistry

Tumor tissues and their matched normal mucosa blocks were obtained from 545 CRC patients (Nanfang Hospital of Southern Medical University, Guangzhou, China). Patients and/or their relatives approved the use of their clinical materials for research purposes according to the Ethics Committee of Southern Medical University. Clinical samples were immunostained with an antibody for PHB (1:500; EP2803Y, Abcam), VEGF (15 μg/mL, AB293NA, R&D) or CD31 (1:400; EPR3094, EPITOMICS). Immunohistochemical staining was performed with the EnVision Detection System (K500711, DAKO Corporation, Copenhagen, Denmark). The immunochemistry results for PHB were evaluated using a previously described method [44]. In each of 10 high magnification fields of every sample, if eccentric distributions or both of concentric and eccentric distributions in any field of a given sample, we defined this case as eccentric distribution; only concentric distribution was observed in any fields, this case was defined as concentric distribution. Interstitial VEGF data were obtained by examining 10 photos for each case using Image-Pro Plus (IPP) version 6.0 (Media Cybernetics, Inc., Rockville, MD, USA).

### Immunofluorescence assay

CRC cells were fixed in 4% paraformaldehyde. The CRC tissue was fixed with acetone for 10 min. Cells and tissues were incubated with PHB (1:100, EP2803Y), PHB (1:20, 4D3G5, Proteintech), CD44(1:40, 15675-1-AP, Proteintech), RhoT1 (1:100, sc-102083, Santa Cruz), COXIV (1:50, 66110-1-Ig, Proteintech), Kinesin (1:300, SUK-4, Abcam), ALDH1 (1:100, Cat#611194, BD), γ-tubulin (1:50, Cat#66320-1-Ig), F-actin (5 μg/mL, 4E3.adl, Abcam), or the microtubule marker (1:100, NM003376, Santa Cruz) antibodies overnight at 4 °C. The cells and sections were stained with secondary antibodies (1:100, Zhongshan Golden Bridge Biotechnology) and nuclei stained for 4’, 6-diamidino-2-phenylindole (DAPI, Sigma) and then cells were counted and examined using confocal microscopy (FV1000, Olympus).

### Analysis of PHB in plasma membrane of CRC tissues

The ratio of plasma membrane to cytoplasmic fluorescence intensity (Ipm/Icyt) in twenty cells of each tissue sample was used to quantitatively assess the extent of membrane-localization of PHB.

### Isolation of the plasma membrane and mitochondria proteins

Plasma membrane extracts were prepared using a Membrane and Cytosol protein extraction kit (Beyotime, Haimen, China), and mitochondria proteins were obtained using a Cell Mitochondria Isolation Kit (Best Bio, China). The extraction of plasma membrane and mitochondria proteins were performed according to the manufacturer's instructions. Plasma membrane and mitochondrial fractions were tested for the plasma membrane marker ATP1B1 (1:500, A5793, ABclonal).

### Lentivirus-mediated small hairpin RNA (Lenti-shRNA) against *PHB*

The Lenti-shRNA vector system (GV115) was constructed, packed, and purified by GeneChem (Shanghai, China), and was used according to the manufacturer's protocol. The stem-loop DNA oligo-nucleotides with the highest knockdown efficiency were 5′-AGCAGAGAGGGCCAGATTT-3′.

### Overexpression of *PHB*

The GV358 *PHB* plasmid and the GV358 vector were purchased from GeneChem (Shanghai, China), and were manipulated according to the manufacturer's protocol.

### Quantitative real-time PCR analysis

Total RNA was extracted from cells and reverse transcribed, and the following specific primers for human *PHB* were designed and used: sense 5′-TGGACAAATGCGACGAACC-3′ and antisense 5′-CCCGCTCACTTGCT GCTT-3′. Gene expression was normalized to *GAPDH*. PCR was performed using Ex Taq™ DNA Polymerase (Takara Bio) and an ABI PRISM 7500 Sequence Detection System (Applied Biosystems). Each sample was tested in triplicate.

### Small interfering RNAs (siRNAs)

The siRNAs against *Miro-1* were a SMART pool of 4 distinct siRNAs, as described by Morlino et al [45], and purchased from Gene Copoeta. Small interfering RNAs (siRNAs) against *NRP1* (5′- GGACAGAGACTGCAAGTAT -3′) or *CDC42* (5′- AAAGACTCCTTTCTTGCTTGT -3′) were purchased from Rib-bio (Guangzhou, China) and were manipulated according to the manufacturer's protocol.

### Migration and PHB re-orientation assays

Cell migration was detected by transwell migration and wound healing assays. For the transwell assay, 2 × 10^5^ cells in 200 μL of media containing 1% fetal bovine serum (FBS) were seeded into the upper chamber (pore size, 8 μm, BD Biosciences). Medium (600 μL) containing 10% FBS with/without VEGF (100 ng/mL, Cat#100-20, Peprotech, USA) was added to the lower chambers. The migrating cells in 10 random fields were counted under a light microscope at 200× magnification.

For wound-healing assays, 10^5^ CRC cells were seeded in each well and then wounded with a 10-μL pipette tip. The cells were incubated with or without VEGF at 37 °C and 5% CO_2_. Images of the cells were obtained at 0 h and 24 h. Cells located within 200 μm of the wound were then examined using confocal microscopy to determine which cells expressed PHB and were faced toward the wound.

### Cdc42 pull-down assay, immunoprecipitation, and immunoblotting

The active Cdc42 Detection Kit (Cell Signaling) was used according to the manufacturer's instructions. Briefly, GTP-bound Cdc42 was isolated with glutathione agarose beads bound to the p21-binding domain of PAK1 via a GST tag, and then identified by immunoblotting of eluted proteins with an antibody for Cdc42.

### Animal model assay

Animal experiments were performed under the guidelines set forth by the Ethics Committee of Medical Research of Southern Medical University, China. For the tumorigenicity assays, tumor cells (2 × 10^6^ cells) were injected subcutaneously into nude mice. The mice were sacrifced 4 weeks after injection. Tumor volume was observed.

### Statistical analysis

The results are expressed as mean ± SD or mean ± SEM. The data were analyzed by Student's t test or one-way ANOVA to determine the statistical significance. Pearson's χ^2^ test or Fisher's exact test was used to analyze the relationship between PHB expression and the clinicopathological features. Survival curves were obtained by the Kaplan-Meier method. All analyses were two-sided and conducted using SPSS version 13.0 for Windows; values of P < 0.05 were considered statistically significant.

## SUPPLEMENTARY MATERIALS FIGURES



## Abbreviations

PHB: prohibitin
CRC: colorectal cancer
VEGF: vascular endothelial growth factor
CDC42: cell division cycle 42
ELISA: enzyme-linked immune sorbent assay
NRP1: neuropilin-1
HUVECs: human umbilical vein endothelial cells
PBMCs: peripheral blood mononuclear cells
CO-IP: co-immunoprecipitation
A7R: ATWLPPR
MTOC: microtubule organizing center
P-cofilin: phosphorylation of cofilin
LIMK: LIM-kinase
