# Supplementary material for: Prohibitin, relocated to the front ends, can control the migration directionality of colorectal cancer cells
Source: Oncotarget. 2017 Jul 19;8(44):76340–56. doi: 10.18632/oncotarget.19394 (PMC5652710; doi:10.18632/oncotarget.19394)
Supplement: Supplementary file 1 [file oncotarget-08-76340-s001.pdf]

Prohibitin, relocated to the front ends, can control the migration directionality of colorectal cancer cells

SUPPLEMENTARY MATERIALS

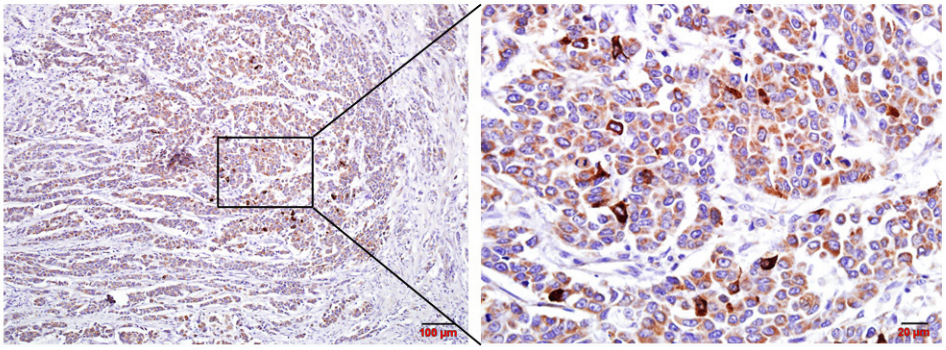

Supplementary Figure 1: Samples lacked intact glands in poorly differentiated adenocarcinoma. Scale bars: 100 µm, and bars in enlarged images: 20 µm.

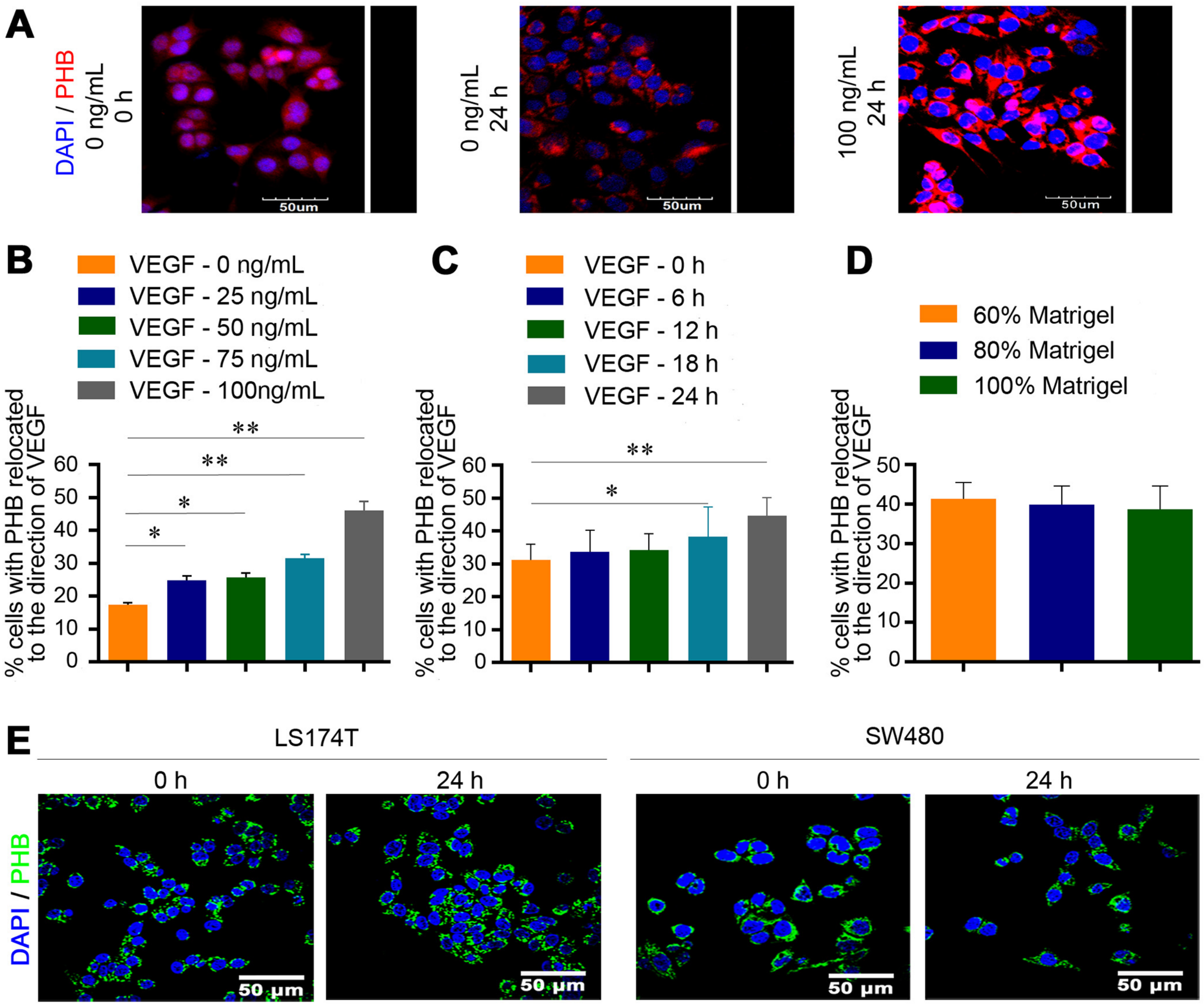

Supplementary Figure 2: Vascular endothelial growth factor (VEGF) stimulation affects PHB localization. **(A)** Wound healing assays were used to assess time-concentration effects in CRC cells with VEGF stimulation. The white lines indicates the direction of the wound. Scale bar: 50 μm. **(B)** PHB relocation depends on the concentration of VEGF. $^*P < 0.01$, $^{**}P < 0.001$. Data are shown as means ± SEM. **(C)** VEGF stimulation time also affects the relocation of PHB in CRC cells. $^*P < 0.05$, $^{**}P < 0.001$. Data are shown as means ± SEM. **(D)** Matrigel concentration does not affect PHB relocation. **(E)** Relocation of PHB in LS174T and SW480 after VEGF stimulation for 24 h. VEGF is placed at upside for chemoattractant. Scale bar: 50 μm.

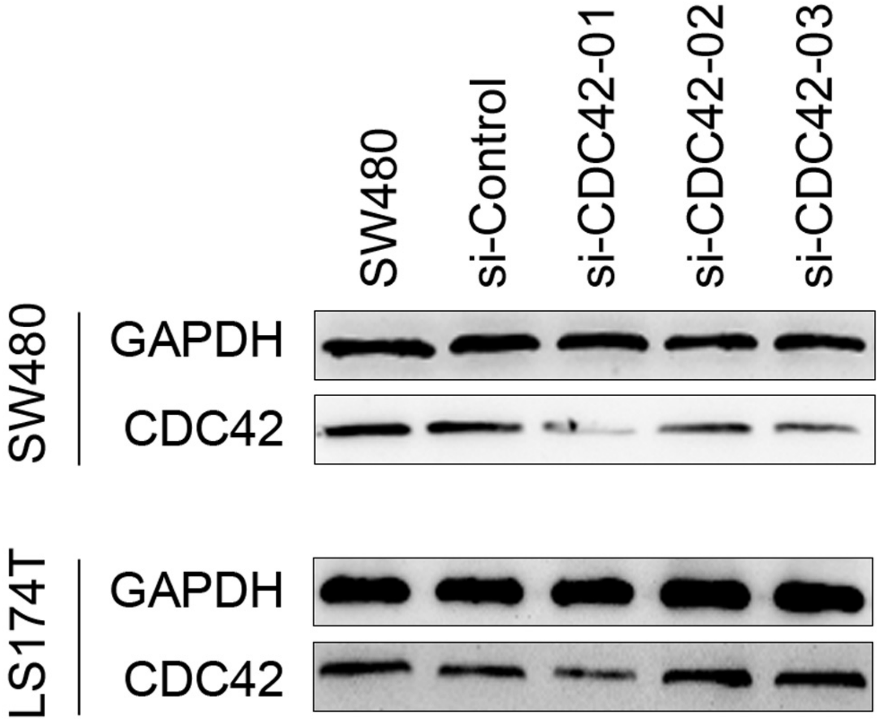

Supplementary Figure 3: Western blot analysis of SW480/LS174T and control CRC cells (si-Control) and CDC42-silenced cells (si-CDC42-01, si-CDC42-02, si-CDC42-03).

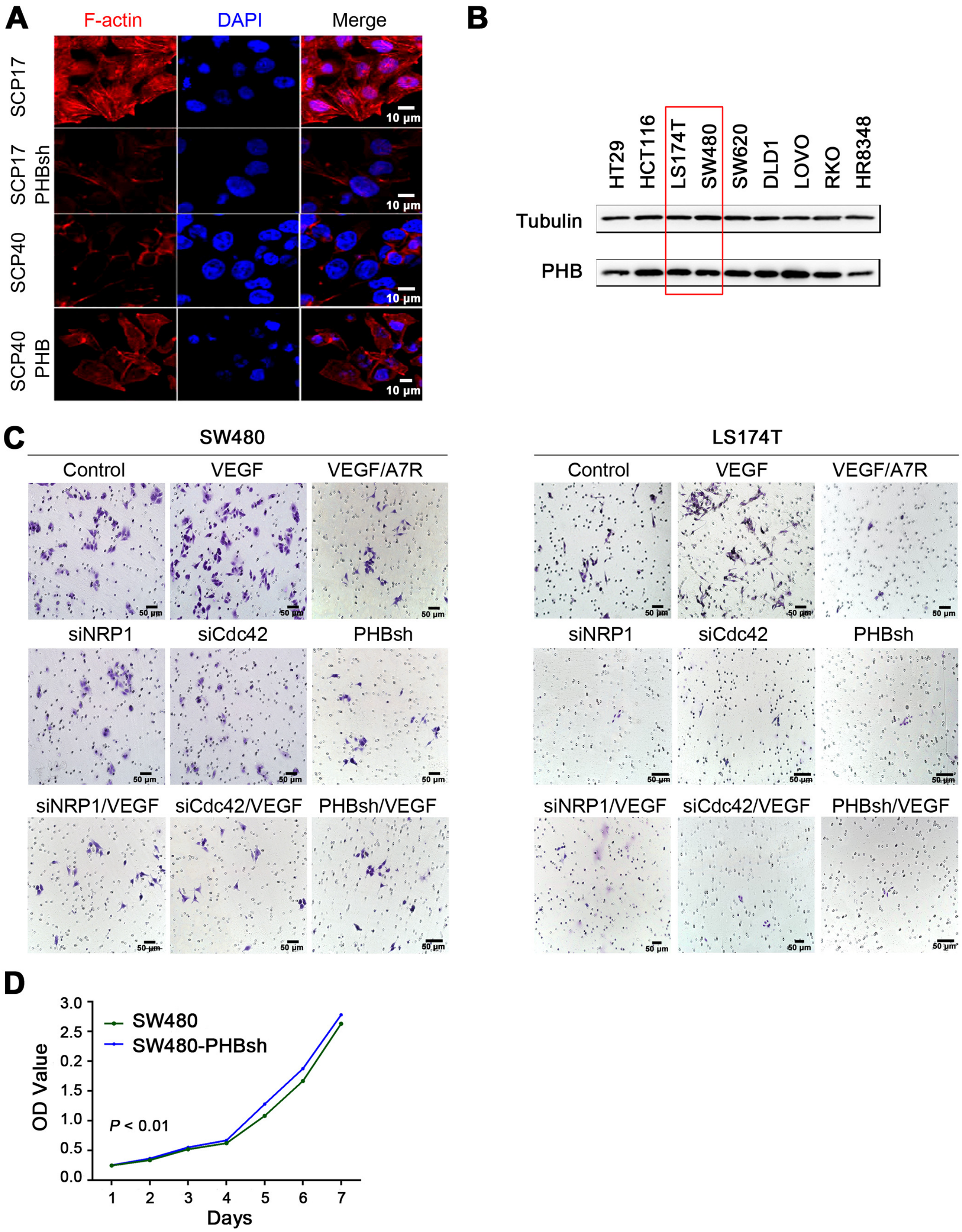

Supplementary Figure 4: PHB influences the cytoskeletal remodeling. (A) Immunofluorescence image of F-actin and PHB co-localization in SCP17, SCP17-PHBsh, SCP40, and SCP40-GV358-PHB (SCP40-PHB) cells. Scale bar: 10 μm. **(B)** Western blot analysis of PHB in CRC cells, red frame indicates the cells moderately expressed PHB among the cell lines. **(C)** Images from a transwell assay for cell migration showed the inhibitory effects of knockdown PHB/Cdc42/NRP1 (PHBsh, siCDC42, siNRP1) and supplemented with A7R (A7R) on VEGF-stimulated migration (hematoxylin staining). VEGF-treated cells (100 ng/mL) migrated into the lower chamber were more than the control. Scale bar: 50 μm. **(D)** Cell growth curve of SW480 and SW480-PHBsh. PHBsh tumor cells showed a bit of high growth *in vitro* after cultured for 4 days, $P < 0.01$.
